# Managing surgical waiting lists through dynamic priority scoring

**DOI:** 10.1007/s10729-023-09648-1

**Published:** 2023-06-28

**Authors:** Jack Powers, James M. McGree, David Grieve, Ratna Aseervatham, Suzanne Ryan, Paul Corry

**Affiliations:** 1 School of Mathematical Sciences, Faculty of Science, Queensland University of Technology, 2 George St, Brisbane, QLD 4000 Australia; 2 Centre for Data Science, Queensland University of Technology, 2 George St, Brisbane, QLD 4000 Australia; 3 Department of General Surgery, Surgical and Critical Care Directorate, Sunshine Coast University Hospital, 6 Doherty Street, Birtinya, QLD 4575 Australia; 4 School of Medicine, Griffith University, 6 Doherty Street, Birtinya, 4575 QLD Australia

**Keywords:** Simulation, Waiting list, Elective surgery, Patient prioritisation, Genetic algorithm, Multi-criteria decision-making, Equity in treatment, Operations research, Operations management

## Abstract

Prioritising elective surgery patients under the Australian three-category system is inherently subjective due to variability in clinician decision making and the potential for extraneous factors to influence category assignment. As a result, waiting time inequities can exist which may lead to adverse health outcomes and increased morbidity, especially for patients deemed to be low priority. This study investigated the use of a dynamic priority scoring (DPS) system to rank elective surgery patients more equitably, based on a combination of waiting time and clinical factors. Such a system enables patients to progress on the waiting list in a more objective and transparent manner, at a rate relative to their clinical need. Simulation results comparing the two systems indicate that the DPS system has potential to assist in managing waiting lists by standardising waiting times relative to urgency category, in addition to improving waiting time consistency for patients of similar clinical need. In clinical practice, this system is likely to reduce subjectivity, increase transparency, and improve overall efficiency of waiting list management by providing an objective metric to prioritise patients. Such a system is also likely to increase public trust and confidence in the systems used to manage waiting lists.

## Highlights

- We propose a dynamic priority scoring system to prioritise elective surgery patients.
- Patient waiting time and explicit clinical factors are incorporated into a mathematical formula to produce a score, which increases over time relative to clinical need.
- Provides objective metric for waiting list staff, improving admission equity, transparency, and efficiency.
- Results suggest this system has potential to reduce subjectivity in clinical decision making, increasing stakeholder’s trust and confidence in surgical waiting lists.

## Introduction

Waiting lists are an inevitable part of the allocation of public health resources when demand for a service is greater than supply. This is particularly evident in the case of managing elective surgery waiting lists. Long waiting times have consistently received media attention due to the negative effects and consequences long waiting times have on the physical and mental health of patients; patients waiting excessive amounts of time for treatment can experience significant impacts on their quality of life and are more susceptible to clinical deterioration, depression, discomfort, and anxiety [1–4].

This problem is set to increase, with the number of Australian public hospital admissions increasing on average by 2.1% per year between 2014 to 2019 [5]. The impacts of COVID-19 have further exacerbated this problem, with admissions decreasing by 9.2% in 2020 from the previous year due to the cancellation of non-urgent surgeries in March 2020 [5]. This created a backlog of patients, thereby further increasing wait times and overall pressure on public health systems [4].

In Australia, patients receiving care through the public health system are assigned a nationally defined urgency classification by their treating clinician, reflecting the recommended timeframe in which the patient should be admitted for surgery. This assessment is based on several clinical factors, such as the patient’s condition, symptoms, and the recommended category assignment for a given procedure according to clinical guidelines [6]. There are three national urgency category classifications with associated maximum recommended waiting times (MRWT), first introduced in the Australian state of Victoria in 1991, and later adopted nationwide [6, 7]:

- Category 1 – Urgent – Procedures that are clinically indicated within 30 days
- Category 2 – Semi-urgent – Procedures that are clinically indicated within 90 days
- Category 3 – Non-urgent – Procedures that are clinically indicated within 365 days

The intent of the three-category system is to differentiate patients with exceptional clinical need or identify those with the greatest potential to benefit from surgery and ensure that these patients are prioritised for care. However, this prioritisation system has been a source of frustration and discontent for a wide range of stakeholders. The criteria used to assign urgency classifications are not clearly defined and are determined implicitly by the treating clinician based on their own experience and training [3]. As a result, variability can exist across clinicians in the classification of patients, negatively impacting the transparency and equity of access experienced by patients [8]. For example, this issue can manifest in the form of ‘category creeping’, where clinicians assign their patients a higher urgency category to progress through the waiting list faster, generally due to a fear that a patient will be disadvantaged by being assessed as non-urgent [7].

While it is accepted that higher priority patients should experience shorter wait times, there is an expectation that patients with similar clinical need should experience comparable waiting times. Due to the lack of clear guidelines that identify key clinical factors to distinguish such patients, extraneous factors such as socioeconomic status or procedure cost-effectiveness may influence the equity of admissions experienced by certain groups of patients [9–11].

### Literature review

There is an urgent need to improve waiting list management of elective surgeries to decrease the negative impacts long waiting lists have on the well-being of patients – especially patients assigned to a lower urgency category – and enhance the transparency and equity of patient prioritisation systems. Many countries around the world such as Canada [12], Italy [13–16], New Zealand [17–19], Spain [20] and the United Kingdom [21–23] have investigated prioritisation systems to varying degrees, with the aim of ranking patients transparently through clear and defensible criteria. These tools have been found to potentially reduce patient waiting times and improve admission equity and consistency [4, 24].

Contemporary patient prioritisation tools are modelled around two fundamental concepts to determine waiting list rank of each patient: scoring systems and formula-based prioritisation tools. Scoring systems identify key clinical factors and develop relative weightings for each criterion in consultation with clinicians and are often additive models, where the summation of the relevant criteria yields the patient’s score [12, 19, 20, 25, 26]. Formulae-based prioritisation systems incorporate waiting time and clinical factors as input into a mathematical formulation to calculate a priority score [13, 14, 16, 21–23]. The intent of these systems is to enable patients to accrue their priority score at a rate relative to their clinical need, while balancing the urgency of other patients on the waiting list.

A notable weakness of scoring systems is omission of waiting time as an explicit criterion. In some implementations, patients may require a minimum score to be considered eligible for surgery. These minimum scores can differ across procedures and are set by the governing health care body, depending on the availability of funding and resources [27]. This rationing technique can be described as rationing through denial, where “care providers turn away would-be patients on the grounds that their needs are not urgent enough” [28]. As waiting time is not an explicit factor in such systems, low priority patients may face excessive waiting times or outright denial of care, and patients must deteriorate sufficiently until they are deemed urgent enough to receive care. This represents a loss of opportunity for pre-emptive care and can potentially lead to further deterioration or emergency room presentation [29].

In formula-based priority systems, the functional form of the prioritisation formula is the defining feature of such a system, dictating how patients progress on the waiting list according to specific criteria. However, in the literature, the functional form can vary greatly, in terms of clinical factor selection, criteria weightings, and other functional coefficients and operators [22, 23]. A selection of early prioritisation formulations to modern day formulations from the literature can be seen in Appendix A, where the range and variety of functional forms is evident.

As such, a notable gap in the literature is the use of rigorously weighted clinical criteria used in conjunction with a time-based prioritisation formula. Although independently, there exists literature on both topics, these two areas have not been thoroughly combined to create an all-encompassing system that enables clinicians to assess the clinical need of patient in a fair and transparent manner, in conjunction with an efficient and equitable means of incorporating this information to manage waiting lists.

### Overview of paper

- Section 2—Aims introduces the motivation and background of this research problem. Specifically, the need to prioritise elective surgery waiting lists more equitably, transparently, and efficiently.
- Section 3 – Methodology describes the approach used to address the research problem, detailing the use of a simulation model to compare the current Australian prioritisation system to the proposed system. This section also describes the development of the clinical factor selection forms (including criteria identification and weighting), data collection, proposed prioritisation formula, and the simulation model (including simulation design, initialisation, calibration). The clinical aspects of the methodology can be found in Sections 3.1–3.3, while the technical implementation of the simulation model can be found in Section. 3.4.
- Section 4 – Results presents the findings and summary statistics of the two simulation models to facilitate comparison in terms of efficiency and equity.
- Section 5 – Discussion & Sect. 6 – Conclusion provide interpretation and analysis of results, as well as clinical implications of this research. Future research directions are also presented and discussed.

## Aims

The aim of this project is to quantify clinical assessment by making explicit the thought processes and common practices clinicians use during patient assessment and provide a clear, transparent, and objective priority metric that can be used to prioritise patients. To do so, this study investigated the use of a prioritisation formula to rank patients, using a simulation model to compare patient waiting time outcomes under the proposed system to the current three-category system.

A time-based prioritisation formula can be generalised in the form of $P=\alpha t$, where $P$ is a patient’s priority score, $\alpha$ is the urgency coefficient which encompasses factors related to a patient's condition, and $t$ is the number of days a patient has been on the waiting list [13]. The intent of such a system is that a patient’s priority score increases at a rate relative to their clinical need and proportional to their waiting time. This facilitates the equitable distribution of access for all patients and ensures that non-urgent and semi-urgent patients are not disproportionally disadvantaged.

A key motivation of this research is that clinical staff of a South East Queensland public hospital identified a gap between the clinical assessment process and the administrative process of booking patients for surgery. When assessing a patient for surgery, clinicians would implicitly identify key factors for a given procedure and use their medical training and experience to assign the patient a waiting list classification in one of three urgency categories based on clinical need. However, non-clinical factors often affect this classification, such as individual patient expression of the pain or inconvenience of their condition, or clinician concern regarding waiting list management.

Once the patient is placed on the waiting list, there is potential for a disconnect to occur between the clinical assessment and the processes used by waiting list staff to manage the waiting list. Waiting list staff are responsible for booking patients into surgery from the waiting list and may not have access to the particulars of every patient. As such, they must rely on the assigned urgency category and other implicit factors (such as waiting time, aggravating factors, clinician preferences, etc.) to prioritise patients and plan surgeries. Additionally, the booking process may require several operational decisions to be made across a number of staff members, which increases variability in decision making due to the number of subjective decisions. This can result in large waiting time discrepancies for patients requiring the same procedure, potentially resulting in patients waiting well past their MRWT. This is demonstrated by the current waiting list of the hospital included in the study – in the fourth quarter of 2022, the proportion of category 1, 2, and 3 patients waiting for surgery was 6.6%, 33.3%, and 60.1%, respectively [30].

## Methodology

A simulation model was developed to assess the impact of a dynamic priority scoring (DPS) system on patient waiting time behaviour compared to the current three-category system. Simulation was chosen as it was determined to be the most appropriate technique to account for the stochastic nature of surgical waiting lists. Simulation has been found to be the most appropriate tool to evaluate the impact of interventions in healthcare management systems, especially in hospital environments where experimentation may not be feasible or cost effective [31, 32]*.* Compared to traditional mathematical optimisation models, simulation is more flexible and can better model the complexities of real-world health systems by incorporating the many sources of variability and stochasticity involved in healthcare operations, such as patient arrivals, resource scarcity and availability, surgical durations, and other randomly occurring events [32, 33]. As such, the use of simulation to model healthcare systems is becoming increasingly popular and has been used extensively in a number of studies to assess the impact of interventions in a variety of healthcare settings, such as prioritisation, waiting list management, scheduling, and demand forecasting [21, 31–37].

The simulation horizon in the model was set at three years, and the patient arrival schedule was generated through resampling historical arrivals from the collected data under the three-category system. A genetic algorithm was used to calibrate the number of daily theatre sessions in the model, so that summary statistics of the simulated data matched summary statistics of the historical data. The three-category and DPS simulation models were then run in parallel, with the results aggregated and compared, providing a retrospective comparison of the two prioritisation techniques.

### Clinical factor selection form development

The first step in developing the DPS system was determining the criteria and associated weights to be used to assess patients and their respective priority coefficient ($\alpha$). In collaboration with clinicians, clinical factor selection forms were developed for ten procedures in the general surgery specialty (breast, cholecystectomy, colectomy, diagnostic laparoscopy, hernia, pancreatic resection, perianal, reversal of stoma, skin lesion excision, thyroidectomy). For each procedure, relevant factors (i.e., criteria) that clinicians would implicitly use to assess patient priority were identified. Two additional factors were included to incorporate relevant unspecified clinical factors or other difficult-to-quantify non-clinical factors, such as the patient’s ability to care for themselves or others, or the expected benefit of surgery [38]. Each of the identified factors generally consisted of three possible severity levels (akin to variations of low, medium, high, etc.), while a small number consisted only of two levels (binary decisions).

Human decision making has a high degree of variability, especially when decisions are made implicitly. A benefit of using such clinical factor selection forms is that by utilising a set of predetermined factors, clinicians can break down difficult and subjective decisions into a series of smaller judgements, thereby reducing variability in clinical decision making across practitioners [39].

Relative weights for the clinical factors and associated levels were determined using the multi-criteria decision-making tool 1000minds [40]. 1000minds is an online decision-making platform that utilises the PAPRIKA algorithm [41] to evaluate relative weights of criteria through pairwise trade-offs. Firstly, the criteria and associated severity levels were entered into the 1000minds platform. The algorithm then elicits weights for each criterion by presenting participants with a choice between two hypothetical patient circumstances (i.e., trade-offs). In a singular trade-off, the same two criteria are presented, however with contrasting severity states. Participants select the trade-off that they deem to be of higher importance. The algorithm adaptively changes the criteria and severity states presented to the participant based on previous responses and terminates when a sufficient number of trade-offs have been completed to directly weight all criteria, or to infer weightings based on the results of previous trade-offs.

**Fig. 1:**
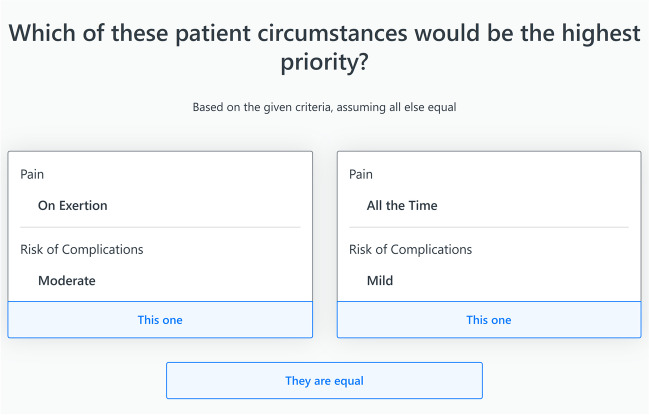
Example of a trade-off choice presented to clinicians for the hernia procedure

Clinicians participated in this trade-off exercise for each of the ten procedures. On average, clinicians required 17 trade-offs per procedure to complete the exercises and determine the final criteria weightings. An example of an initial trade-off for the hernia procedure can be seen in Fig. 1, where two criteria are presented with contrasting severity levels, as determined by the PAPRIKA algorithm. Clinicians selected the circumstance they deemed to be of higher priority, based only on the criteria shown. As clinicians progress through the exercise, the PAPRIKA algorithm adaptively changes the criteria and severity states presented next, based on previous responses. On completion of all trade-offs, final weightings for each of the criteria and associated levels could be determined.

**Fig. 2:**
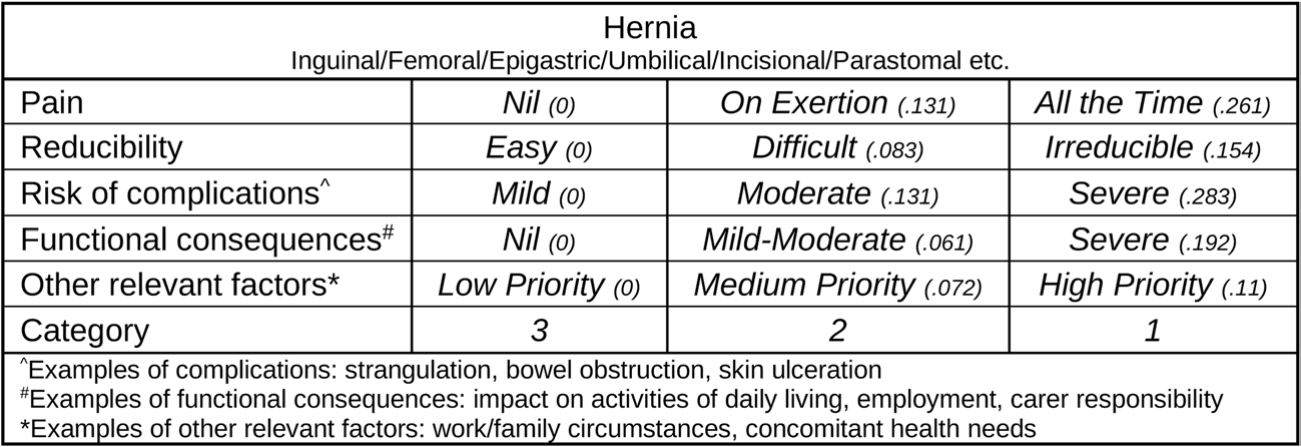
Example clinical factor selection form for a hernia procedure with relative weights for each level of the clinical factors

Figure 2 illustrates an example of a clinical factor selection form for the Hernia procedure, presenting the relevant criteria identified for this procedure in conjunction with the associated criteria weightings calculated through the trade-off exercises, as described above. These forms were utilised by clinicians on the project team during the data collection phase of the study (described below in the following section) to collect clinical factor data of historical patients. Rows represent criterions while columns represent criterion severity. The category criterion provided clinicians with the opportunity to revise the assigned urgency category of a patient if they deemed the original assignment was inappropriate.

After reviewing historical case files, clinicians select one box from each row that most accurately describes the condition of the patient under review. Selecting the left most box of each criterion would yield a combined factor score of zero, while selecting the right most box of each would yield a factor score of one. Hence, any combination of clinical factors yields a factor score between zero and one. Weights for each criterion level are only provided for the reader’s reference in Fig. 2 and are not included in practice to avoid clinician manipulation. The full list of general surgery procedures included in this study with associated clinical factors, sub-levels, and weights, can be found in Appendix C.

### Data collection

Data were collected on patients placed on the waiting list for an eight-month period in 2019, comprising a cohort of approximately 850 general surgery patients for the ten predefined procedures. The proportion of categories 1, 2, and 3 patients collected during this period was 53%, 39%, and 8%, respectively. Data fields included assigned urgency category, procedure, date added to the waitlist, date of surgery and surgical durations (such as procedure length and total operating theatre use). All collected data were complete, except in a small number of instances where surgical duration data were missing or erroneous. These durations were replaced with values sampled from a log-normal distribution [42], fitted to the historical surgical duration data.

Clinical factor data were collected by clinicians from the project team, who reviewed historical case data for each patient through the hospital’s electronic record management system, before completing the appropriate clinical factor selection form. As mentioned above, clinicians also had the opportunity to revise the assigned urgency category if they deemed the original assignment was inappropriate.

**Fig. 3:**
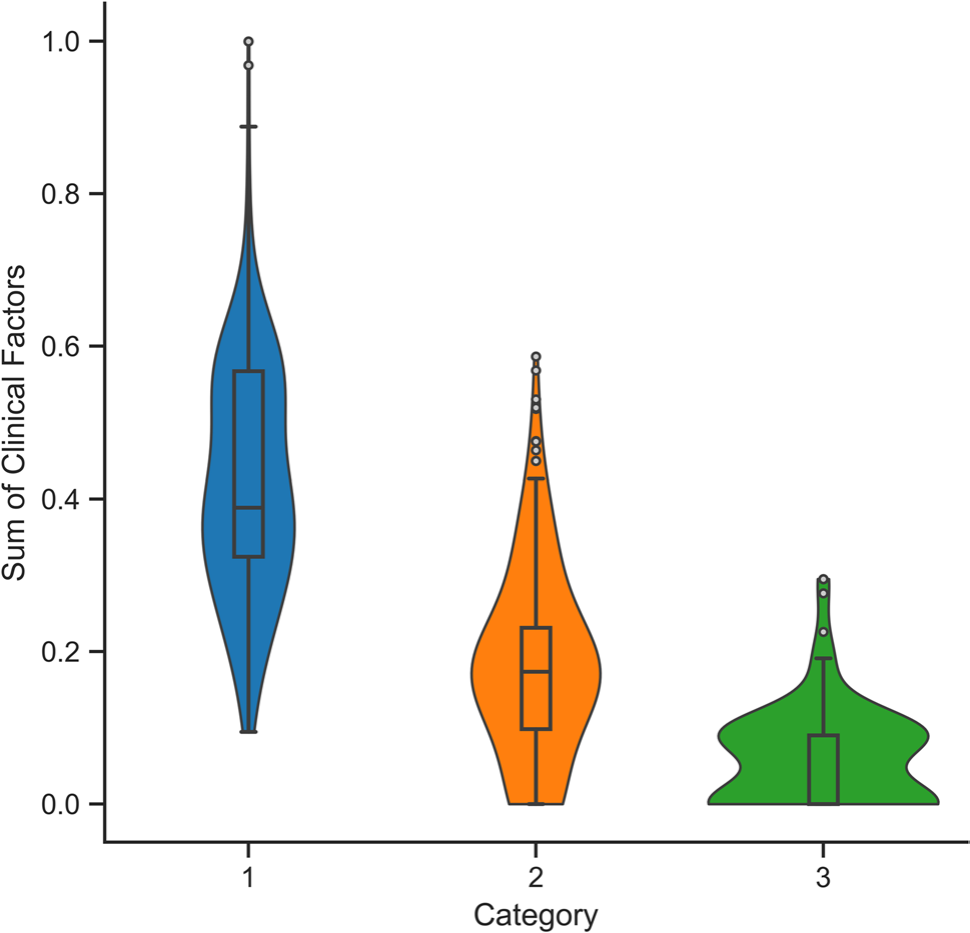
Clinical factor score distribution by category

Full summary statistics across a variety of waiting time metrics collected from the historical data can be seen in Table 1, while a summary of the clinical factor data collected for each patient, grouped by urgency category, can be seen in Fig. 3. In this figure, a relationship is evident between urgency category and factor score, where higher priority patients tend to score higher. This relationship ensures that patients can be adequately differentiated in terms of clinical urgency based on their factor score. Patient and clinical factor data used in this study has been made available online [43].

**Table 1:** Summary statistics of patient waiting times in days (proportion of MRWT) for historical data, three-category and DPS simulation

	CATEGORY	COUNT	MEAN	STD	5%	Q1	MEDIAN	Q3	95%
Historical	1	428	25.13 (0.8375)	11.72 (0.3905)	9 (0.3)	19 (0.6333)	25 (0.8333)	29 (0.9667)	42.65 (1.4217)
	2	334	87.29 (0.9699)	35.79 (0.3977)	42 (0.4667)	69.25 (0.7694)	84 (0.9333)	98 (1.0889)	154.75 (1.7194)
	3	84	364.98 (0.9999)	52.87 (0.1448)	300.9 (0.8244)	333.75 (0.9144)	360.5 (0.9877)	394.25 (1.0801)	450.65 (1.2347)
Three-category*	1	30,243	25.32 (0.8439)	7.06 (0.2353)	12 (0.4)	21 (0.7)	26 (0.8667)	28 (0.9333)	35 (1.1667)
	2	21,268	85.25 (0.9473)	23.32 (0.2591)	59 (0.6556)	68 (0.7556)	82 (0.9111)	101 (1.1222)	129 (1.4333)
	3	3848	364.28 (0.998)	69.48 (0.1904)	292 (0.8)	327 (0.8959)	362 (0.9918)	399 (1.0932)	474 (1.2986)
DPS*	1	30,200	27.69 (0.9229)	7.38 (0.2461)	17 (0.5667)	24 (0.8)	28 (0.9333)	33 (1.1)	40 (1.3333)
	2	21,156	84.72 (0.9413)	21.56 (0.2395)	49 (0.5444)	75 (0.8333)	84 (0.9333)	98 (1.0889)	117 (1.3)
	3	4059	330.56 (0.9057)	64.5 (0.1767)	242.8 (0.6652)	304 (0.8329)	336 (0.9205)	367 (1.0055)	416 (1.1397)

^*^ Statistics calculated from patients treated within the bounds of the warm-up and cool-down interval

The proportion of patients on the waiting list that were treated within their MRWT is termed the ‘treat in time’ proportion (‘time’ referring to MRWT). This is a key metric used to determine elective surgery performance across hospitals in Australia. A patient can be considered ‘treated in time’ if the result of dividing the number of days a patient has been waiting by their MRWT for their urgency category is less than or equal to one. For categories 1, 2, and 3 patients in the historical data, the proportion of patients treated in time was 77.57%, 60.78%, and 51.19%, respectively. Across all urgency categories, the treat in time proportion was 68.32%.

### Prioritisation formula functional form

We propose the following functional form of the patient prioritisation formula, which focuses on the incorporation of three main principles to calculate a patient’s priority: how long the patient has been waiting; score of clinical factors; and MRWT. The latter two principles encompass the priority coefficient. The priority score of a patient was calculated using the following formula:

$$P=\frac{t}{M}\left[1+\sum_{i=1}^{5}{c}_{i}\right],$$

where $P$ is priority score, $t$ is time on the waiting list in days, $M$ is the MRWT in days for the patient’s assigned urgency category such that $M\in \{30, 90, 365\}$ (i.e., category 1 equates to a MRWT of 30 days, etc., as described in Sect. 1), and ${c}_{i}$ is the associated weight of clinical factor $i$ such that $i=1,...,5$ and $\sum_{i=1}^{5}{c}_{i}\in [\mathrm{0,1}]$. This formulation ensures that a patient’s priority score increases over time at a rate proportional to their clinical need. MRWT ($M$) dictates the overall rate at which a patient’s score increases, while the sum of clinical factors ($\sum_{i=1}^{5}{c}_{i}$) serves as a multiplier to reflect a patient’s clinical need and differentiate them from other patients within the same urgency category. The ratio of $\frac{t}{M}<1$ indicates that the patient was treated within their MRWT.

### Simulation design

A simulation model was developed and calibrated based on historical data to compare waiting time behaviour and patient outcomes under the current three-category versus the DPS system. Comprehensive detail of simulation design, technical implementation, and calibration can be found in Appendix B. This section summarises the information presented in the aforementioned appendix and provides an overview of the techniques utilised to initialise, calibrate, and compare the simulation models.

To provide an analogous comparison to current systems used to prioritise elective surgery patients, an initial simulation model of the current three-category system was developed. This model was based on a classical priority queue, modified accordingly to ensure the behaviour of the model was representative of current practices used by bookings staff to manage the waiting list. This included incorporating certain constraints and tuneable parameters in the model that are not explicitly defined in practice, however, were implemented as simple rules to approximate the complex manual decision-making process surgical booking staff face.

Historical waiting time data were used to calibrate these constraints and model parameters to ensure the developed model was an accurate representation of reality. A combination of appropriate metrics were used in conjunction with several thousand simulation replications to determine optimal values for these parameters. This resulted in a tuned model that closely matched historical behaviour and trends, providing a robust means of comparison to the DPS system.

To generate a patient cohort for each simulation replication, bootstrap sampling from historical patient data was applied. This consisted of randomly selecting patients from historical data each simulation month (with associated attributes, such as procedure, surgical durations, clinical factors, etc.) and appending them to the simulation waiting list. Historical patient data were used to generate an empirical distribution of monthly waiting list arrivals for each urgency category. This distribution was then sampled each month in the three-year simulation horizon to determine the number of patients from each category arriving to the waiting list. These arrivals were subsequently distributed throughout the month to ensure approximately the same number of arrivals each week.

Daily patient throughput in the simulation model was determined by the theatre schedule, which dictates the number of operating sessions per day in the simulation horizon. Each session is four hours in duration. The number of patients that can be scheduled in a session is dependent on the required theatre time for each patient, which encompasses surgery time and changeover time, and was predetermined on commencement of the simulation. In both the three-category and DPS systems, if patients are equal in priority in any regard, prioritisation takes place in descending order of theatre time to ensure patients with long surgical durations are not disadvantaged, due to the decreased flexibility in scheduling long-duration procedures.

While any theatre schedule could be generated randomly and used repeatedly, the use of such a schedule may not produce realistic simulation results that are representative of historical data. Therefore, calibration was necessary to ensure that the simulation was representative of historical behaviour. As such, for each simulation replication, the theatre schedule for the generated patient population and arrival stream under the three-category system was calibrated according to historical data and admission trends. This was achieved by utilising a genetic algorithm implemented using the Python package PyGAD [44], to evolve the optimal number of daily theatre sessions to best match historical performance.

Genetic algorithms (GAs) are a type of evolutionary algorithm inspired by the concepts of evolution, natural selection, and survival of the fittest [45]. The algorithm starts by generating a certain number of candidate solutions, known as a population. Each candidate solution is represented by a vector of fixed length, with each vector denoted as a gene. A fitness score is used to determine the quality of each candidate solution. In each successive generation, new solutions are generated by selecting a number of parent individuals with the highest fitness values and applying perturbation operators to generate offspring individuals. After a predetermined number of iterations, a solution space of high-quality solutions will ideally be achieved [46]. The full methodology and implementation of the GA utilised in this research can be found in Appendix B—Theatre schedule calibration.

The fitness function was developed to minimise the sum of the squared error of four metrics between historical and simulated waiting times for each category, by manipulating the daily number of sessions. The four metrics that were incorporated in the fitness function were the mean, median, first quartile, and third quartile of proportion of MRWT (the normalised equivalent of days waiting according to the patient’s urgency category).

On completion of the genetic algorithm, a local search algorithm was implemented to further improve the evolved solution. This algorithm iterated over all genes randomly in the final solution and added or subtracted one session with equal probability. The fitness of each solution was evaluated and if a higher quality solution was produced, it would be accepted as the incumbent solution.

To provide a high degree of confidence in the results of the simulation, approximately 2000 simulation replications were conducted. A single simulation replication consisted of sampling a new patient cohort across the simulation horizon and calibrating the number of theatre sessions under the three-category system. The DPS system was then run in parallel holding all else equal. However, the quality of the solutions (i.e., the generated theatre schedules for an associated patient cohort) varied dramatically, hence only the top 1% of solutions were included in the analysis. These solutions represented the simulation replications where summary statistics of the three-category simulation model were closest to summary statistics of the historical data, thereby providing confidence in the comparability of the DPS model to historical system behaviour. These top solutions were then aggregated and compared.

## Results

During the simulation horizon of three years, 60,630 patients were simulated over 20 simulation replications (encompassing the top 1% of generated patient cohort and theatre schedule pairs), with the results aggregated. Due to the imposed warm-up and cool-down periods, 55,359 (91.3%) and 55,415 (91.4%) patients were included in the analysis for the three-category and DPS systems respectively. This difference can be attributed to the prioritisation methodology of the two systems; for example, a patient admitted to surgery within the bounds of the warm-up and cool-down periods of one prioritisation system may not necessarily also be admitted under the other system within the same bounds. Waiting time metrics for the simulation results of the three-category system compared to historical data can be seen in Table 1. It can be seen in the aforementioned table that the genetic algorithm replicated historical metrics and trends closely, providing assurance on the comparability of results.

### DPS system behaviour

**Fig. 4:**
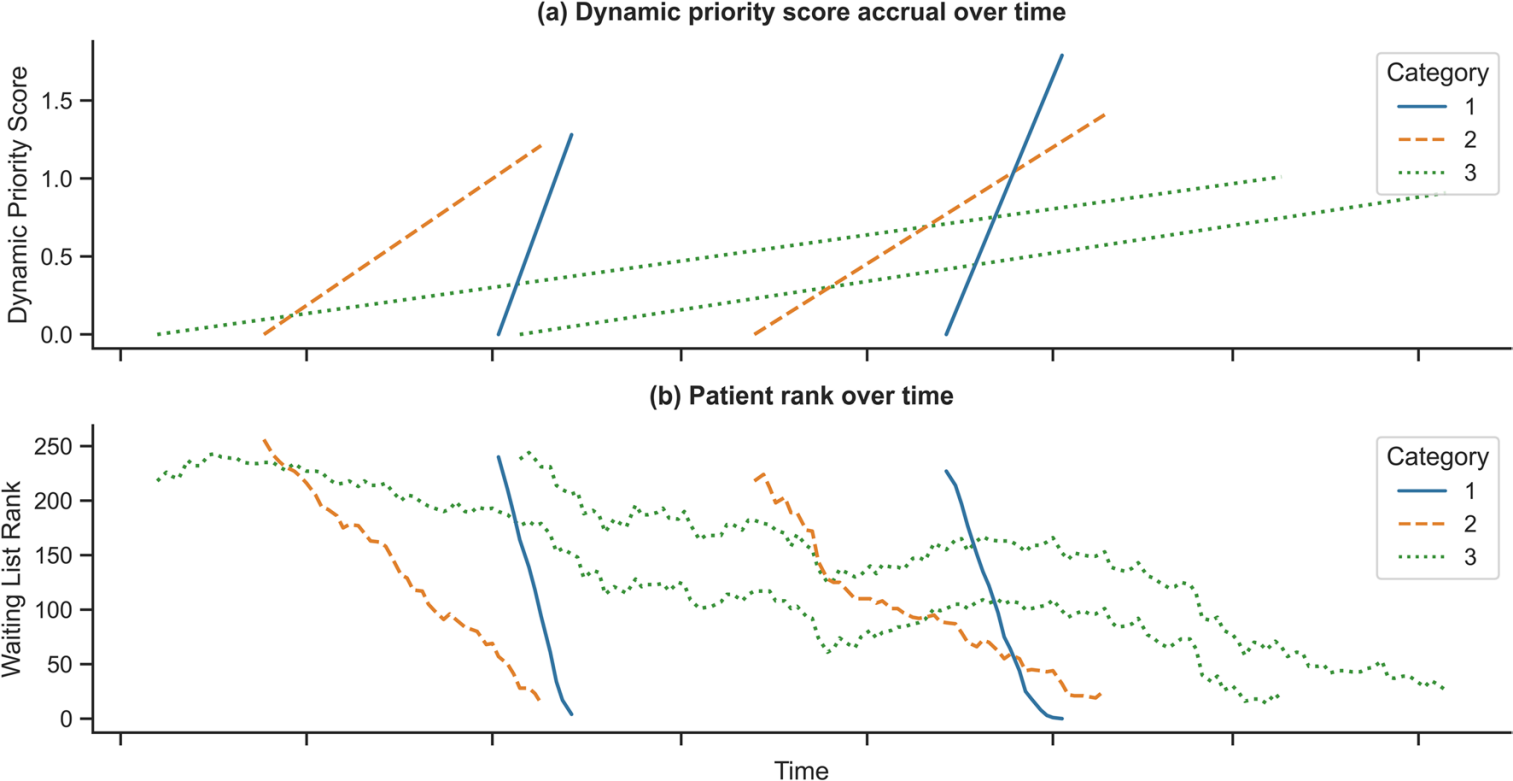
Priority score accrual (**a**) and patient rank (**b**) over time for selected patients

Utilising a DPS system implements a waiting list with a single queue, where patients ascend in priority according to their score. Priority score accrual for a selection of patients across the three urgency categories can be seen in Fig. 4(a). Given that a patient’s priority score can ascend at different rates, a patient’s rank on the waiting list can fluctuate. Under the DPS system, there was an overall tendency for patient rank to decrease over time, regardless of urgency category. This behaviour can be seen in Fig. 4(b), where patients of varying priority may change in rank to accommodate higher priority patients or new patients joining the waiting list, while maintaining an overall tendency to progress to the front of the waiting list.

### Absolute waiting time differences

**Fig. 5:**
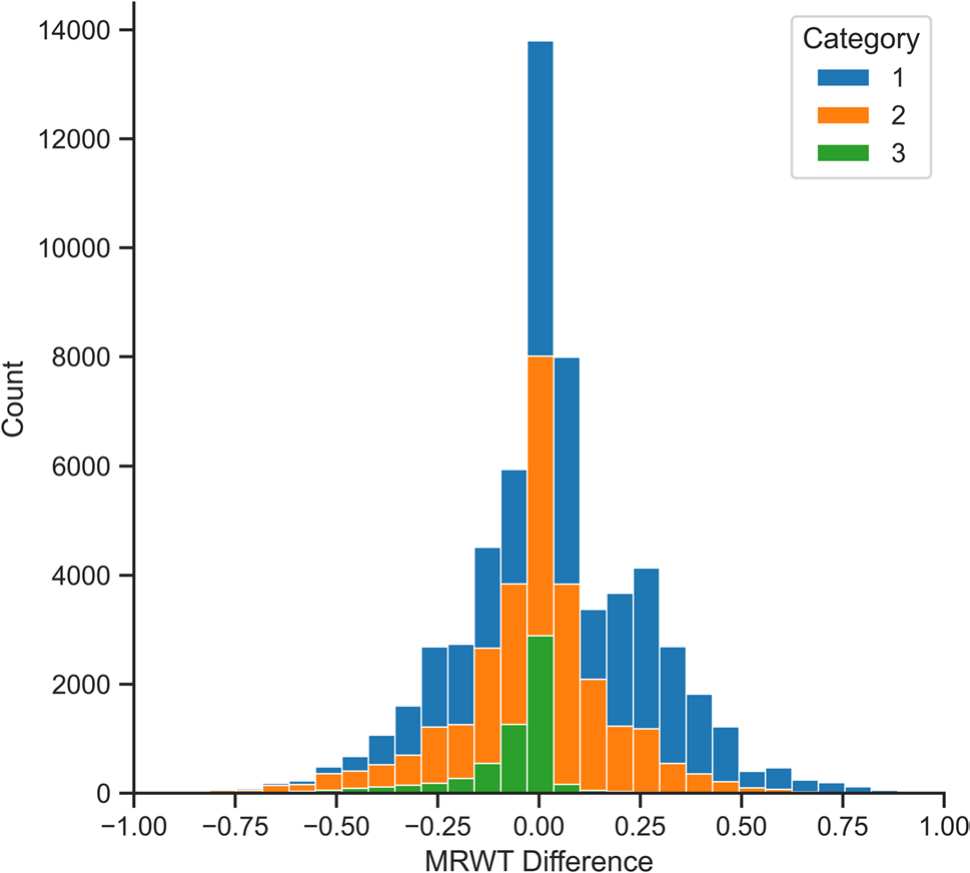
Waiting time differences under the three-category and DPS systems

Under the DPS system, it was found that on average, category 3 patients waited 26.5 days shorter compared to the three-category system (95% CI 25.09–27.91, *p* < *0.0001*). Conversely, category 1 patients on average waited 2.32 days longer (95% CI 2.24–2.4, *p* < *0.0001*). The average waiting time of category 2 patients decreased by 0.61 days; however, this change was not statistically significant (95% CI 0.36–0.85, *p* > *0.05*). The distribution of waiting time differences between the two prioritisation systems can be seen in Fig. 5, where negative values indicate a patient has been admitted sooner under the DPS system and vice versa for positive values. These waiting time differences are demonstrated by the skew present in the aforementioned figure, especially for category 1 and 3 patients.

### Waiting time differences as proportion of MRWT

The improved admissions consistency of the DPS system is demonstrated through the proportion of MRWT metric. This is calculated to be the time waited divided by the respective MRWT for that patient’s assigned urgency category. As this is a normalised metric, it is valid to use it to compare all patients, regardless of urgency category – hence, this metric can also be used considered as a measure of waiting time equity.

**Fig. 6:**
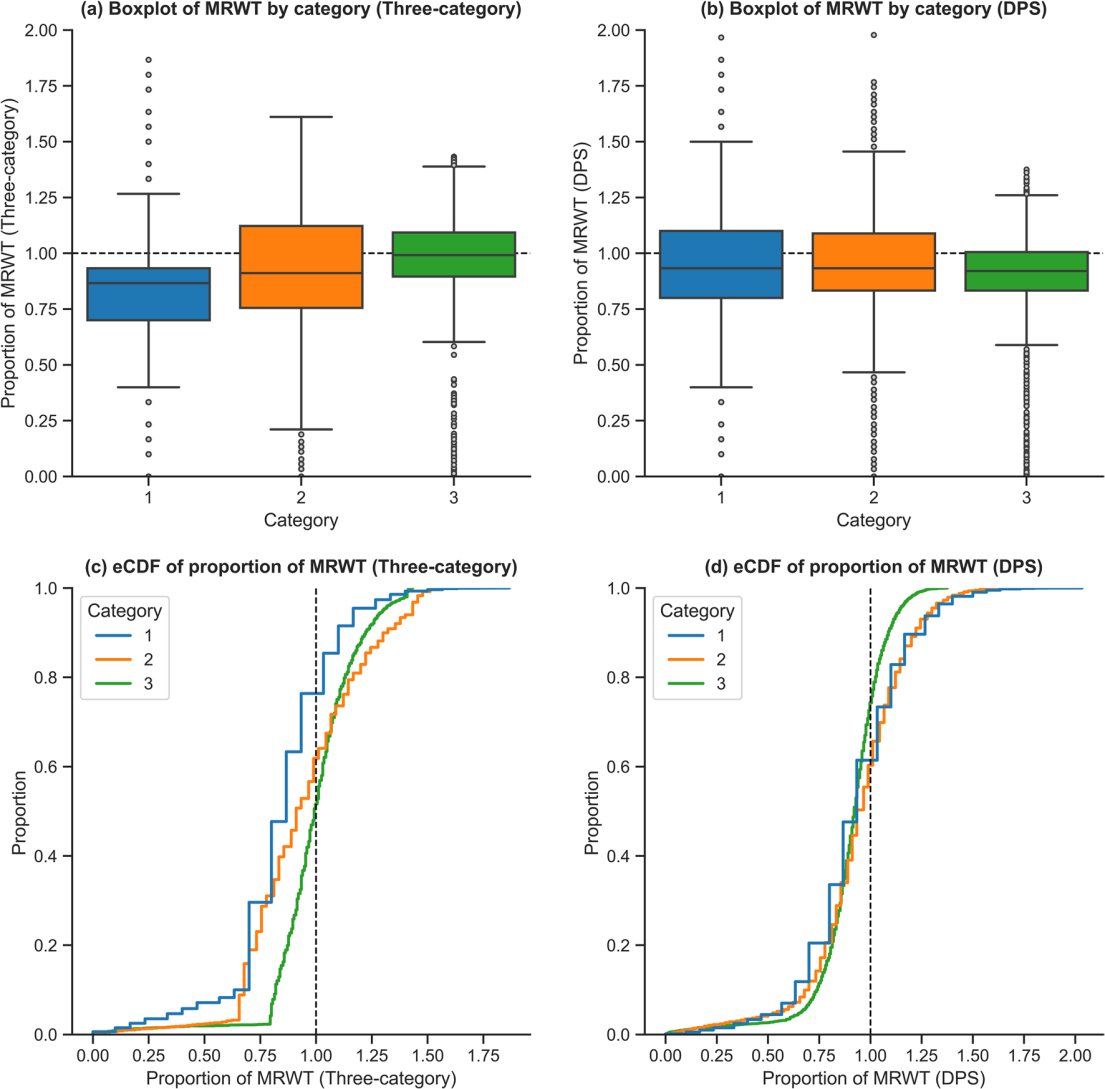
Comparison of proportion of MRWT and empirical cumulative density function of proportion of MRWT between the three-category and DPS systems

As such, Fig. 6(a) demonstrates category 1 patients under the three-category system wait a much lower proportion of MRWT (median of 86.7% of MRWT) compared to category 2 & 3 patients (median of 91.1% and 99.2% of MRWT, respectively). Given this metric is normalised, category 1 patients can be considered to be disproportionately advantaged.

Conversely, Fig. 6(b) demonstrates that under the DPS system, patients across urgency categories waited approximately the same proportion of their MRWT. Under this system, the median waiting time for category 1, 2, and 3 patients converged much closer, to 93.3%, 93.3%, and 92.1% of MRWT, respectively. Additionally, under the DPS system, the interquartile spread of proportion of MRWT decreased for category 2 and 3 patients, while only slightly increasing for category 1 patients. This behaviour can be attributed to patients being explicitly prioritised by this metric in the DPS system, resulting in more consistent admission behaviour and standardised waiting times across all urgency categories. Further metrics comparing these two systems can be found in Table 1.

### Treat in time proportion

As introduced previously, the treat in time proportion is a measure of the proportion of patients that were treated within the clinically recommended time for their urgency category. For categories 1, 2, and 3 patients, the proportion of patients treated in time under the simulated three-category system was 76.38%, 61.84%, and 51.64% respectively, matching very closely with the historical data of 77.57%, 60.78%, and 51.19%. Aggregated across urgency categories, the treat in time proportion was 69.07%. However, under the DPS system, the treat in time proportion decreased to 61.43% and 60.29% for category 1 and 2 patients and increased to 74.28% for category 3 patients. Aggregated across urgency categories, the treat in time proportion decreased to 61.94%.

This behaviour can be attributed to the nature of the DPS system, which admits patients on the basis of proportion of MRWT waited. Effectively, the DPS system exchanges the number of patients treated in time in favour of improved admission consistency across categories. This can be seen in the empirical cumulative density function plot of proportion of MRWT in Fig. 6(c & d), where a tighter distribution under the DPS system is evident, demonstrating increased uniformity and consistency across urgency categories.

### Patients treated earlier/later under the DPS system

**Fig. 7:**
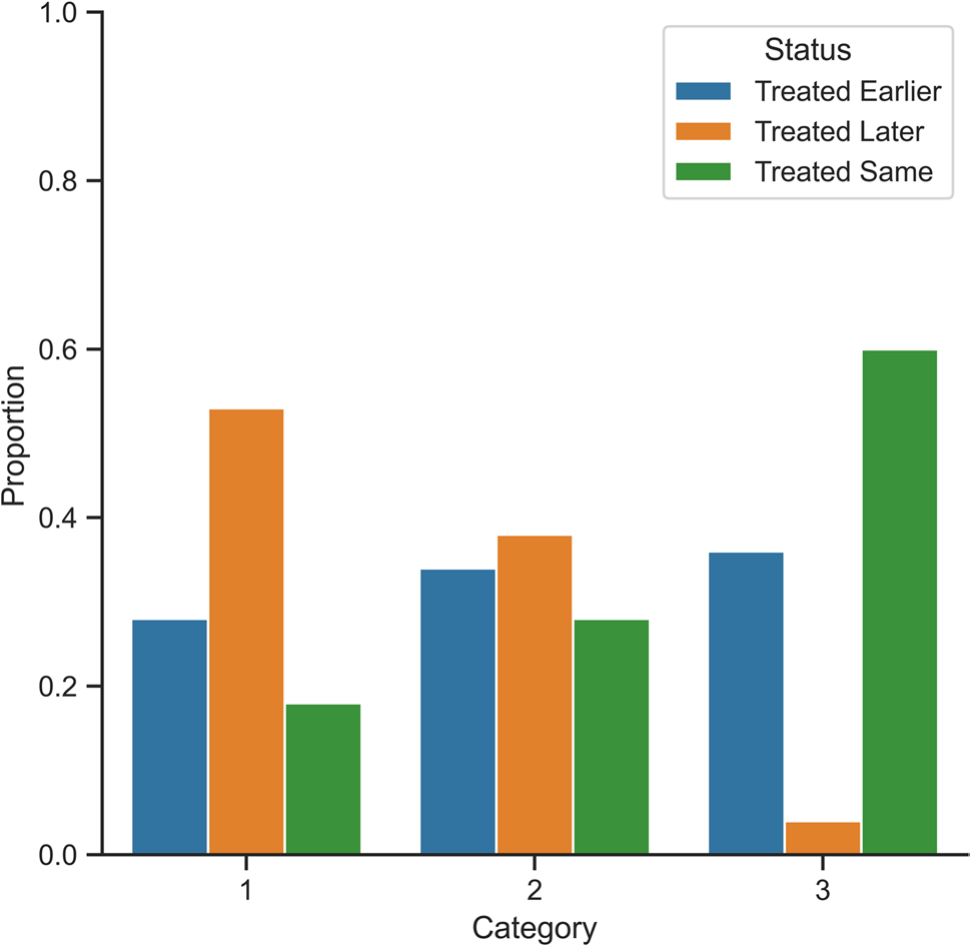
Waiting differences relative to the DPS system

While on aggregate, there was found to be statistically significant differences in waiting times across urgency categories under the DPS system, this may not reflect the true number of patients affected under this system. Overall, it was found that 33% of patients were treated earlier under the DPS system, 44% later, and 23% were unaffected (patients deemed to be unaffected if the change in waiting time was less than 5%). Most category 1 patients were treated later under the DPS system, with 28% treated earlier, 53% later, and 18% unaffected. For category 2 patients, there was a roughly even distribution, with 34% of patients treated earlier, 38% later, and 28% unaffected. The largest proportion of category 3 patients were unaffected by the DPS system, with 36% treated earlier, 4% later, and 60% unaffected. These proportions can be seen visualised in Fig. 7.

### The effect of urgency category and clinical factors on waiting time

**Fig. 8:**
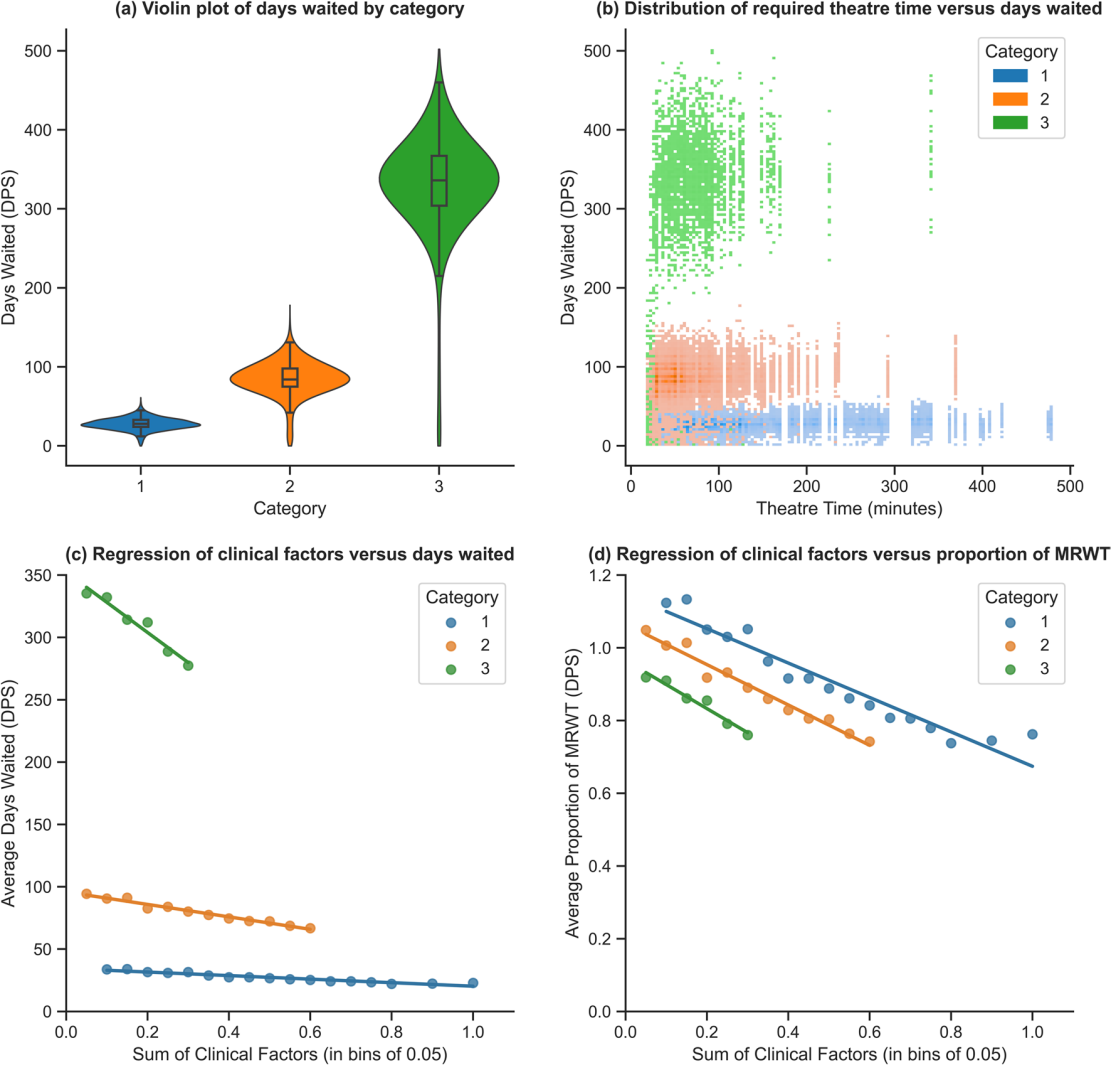
Summary plots of simulation results

It was found that MRWT is a dominating term in the functional form of the prioritisation formula. This is evident from the results of a Tukey’s range test, in which it was found there was a significant statistical difference in waiting time between urgency categories (*p* < *0.0001*). This is visualised in Fig. 8(a), demonstrating not only the difference but also the distribution of waiting time between categories. Figure 8(b) plots the relationship of waiting time in days under the DPS system versus theatre time utilised in minutes. The inclusion of this figure is to support the interpretation of the long tail of category 3 patients as presented in Fig. 8(a) and will be discussed in Sect. 5 – Discussion.

There was found to be a negative linear relationship between clinical factor score and patient waiting time. Due to the volume and variability in the data, each patient’s clinical factor score was first binned into groups of 0.05. The data were then aggregated by bin and urgency category, taking the average of both days and proportion of MRWT waited for each bin. The results of this can be seen in Fig. 8(c) and (d), demonstrating the same linear relationship scaled according to days and proportion of MRWT. Through an ordinary least squares regression, it was found that each of the calculated regressions fitted the data well, with ${R}^{2}$ values of 0.9254, 0.9704, and 0.9533 for category 1, 2, and 3 patients, respectively. The equations for each of these regressions can be seen in Table 2. The intercept term of each equation can be interpreted as the average waiting time for a patient with a factor score of zero (i.e., no aggregating clinical factors) in their respective urgency category. The gradient term can be interpreted as the average decrease in waiting time (subtracted from the value of the intercept) for a patient with a factor score of one (i.e., all aggregating clinical factors). As such, if a patient’s clinical factor is greater than zero, their average wait time decreases according to the multiplicative result of the gradient coefficient and their clinical factor score.

**Table 2:** Regression equations of relationship between average waiting time (y) and clinical factor score (x) for each urgency category

Category	Equation of regression line (days)	Equation of regression line (proportion of MRWT)	${R}^{2}$
1	$y = -14.193x + 34.418$	$y = -0.4731x + 1.1473$	$0.9254$
2	$y = -49.935x + 95.827$	$y = -0.5548x + 1.0647$	$0.9704$
3	$y = -241.31x + 352.24$	$y = -0.6611x + 0.9651$	$0.9533$

## Discussion

Prioritisation techniques have been used extensively to ensure that patients receive timely treatment in a manner that is fair, equitable and transparent. This research demonstrated that by utilising a DPS system that prioritises patients according to explicit clinical factors through a time-based prioritisation formula, the equity experienced by patients can be improved.

The clinical factor selection forms enabled clinicians to assess patients with explicit criteria, providing uniformity on the criteria used in clinical decision making for the procedures within the scope of this study. The weighting of each criterion was also calculated following a mathematically rigorous method, allowing for clinical expertise and experience to be accurately translated into normalised quantitative data.

The DPS system improves equity and consistency by ensuring that certain groups of patients are not disproportionally advantaged or disadvantaged through a transfer of equity, especially between category 1 and 3 patients. For example, it was found that on average, with category 1 patients waiting approximately 2 days longer, category 3 patients could be admitted almost 27 days sooner. This transfer of equity is further demonstrated in Fig. 5 by the distribution of category 1 patients being right-skewed, while category 3 patients were left-skewed. However, patients treated under the DPS system were still treated at approximately the same proportion of their MRWT, demonstrating a more equitable admissions system. Additionally, by prioritising all patients according to a standardised prioritisation formula that incorporates clinical need, better waiting time consistency can be achieved for patients of the same category and similar clinical need.

The use of clinical factors in the DPS system enabled patients of the same urgency category to be differentiated based on clinical need, as demonstrated in Fig. 8(c & d) and characterised by the linear equations presented in Table 2. It is evident that the use of such clinical factors under the DPS system had a tangible effect on waiting time and enabled patients to progress through the waiting list at a rate relative and proportional to their clinical need. Though, it is important to observe that each regression equation is only valid for clinical factor scores within the respective distribution for that urgency category, as presented in Fig. 3.

In each of the equations presented in Table 2, is interesting to note that the intercepts of each equation are approximately equal to the MRWT for the respective urgency category. This suggests that even when demand exceeds supply, using proportion of MRWT to prioritise patients ensures an equitable outcome for patients at all levels of clinical urgency. The use of these equations in clinical practice could enable clinicians and waiting list staff to provide patients with an accurate and up-to-date indication of when they can expect to enter surgery. By continually updating these equations as new patients are admitted, an increased level of transparency could be achieved that perhaps could not be possible under current prioritisation methodologies.

While a transfer of equity was observed between urgency categories, Fig. 8(c & d) demonstrates that a transfer of equity is also present within categories. This can be attributed to the significant effect factor score has on waiting time for patients of all urgency categories. As such, it is not expected that the DPS system would negatively impact high priority patients’ quality of life or mortality in any significant way. In circumstances where a truly urgent category 1 patient is added to the waiting list, this would be reflected by their factor score and therefore, the DPS system would appropriately expedite them. It is only the patients that can afford delay surgical intervention a small proportion of MRWT longer, as indicated by a lower factor score, that would be affected by this transfer of equity. Though, the additional waiting time would be minimal, and in the case of category 1 patients, may equate to approximately 1–2 days. Additionally, given the median factor score for category 1, 2, and 3 patients in the historical dataset were 0.389, 0.174, and 0.09, respectively, this indicates that there is sufficient range to adequately identify patients of extremely high priority through their factor score. Therefore, it can be concluded that the DPS system is capable of equitably prioritising patients of all severity levels while ensuring those of higher priority receive appropriate care without undue risk to quality of life and mortality.

It was found that the summary statistics of patient waiting time for category 3 patients under the DPS system did not reflect the number of patients affected by this prioritisation policy. The proportion of category 3 patients unaffected (60%) under the DPS system suggests that the system itself does not have a broad effect on all patients, but on a select proportion who would have been disproportionally affected under the three-category system; while the three-category system could effectively prioritise high priority patients, it did not adequately prioritise those approaching or those who have waited well past their MRWT. It is perhaps this disproportionately affected group that may have skewed summary statistics. This demonstrates that the DPS system benefits patients by explicitly prioritising according to the proportion of MRWT to ensure consistent wait times across categories.

It was also interesting to note the long tail of category 3 patients in Fig. 8(a), which indicates a small number of patients entering surgery well before their due date. These perhaps represent instances where there was insufficient theatre time in a day to admit any category 1 or 2 patients, but opportunistically, there were category 3 patients with sufficient theatre durations to allow them to access surgery early. This phenomenon can be further demonstrated in Fig. 8(b), where there are a number of category 3 patients who receive treatment much earlier compared to other patients of the same category, with the differentiating factor of much shorter theatre durations relative to patients across all urgency categories.

The use of a DPS system in clinical practice has the potential to reduce subjectivity in clinical decision making and improve consistency in waiting times, as well as improve the overall efficiency of managing the waiting list. The DPS system provides bookings staff with a clear metric on which patients can be ranked and compared. Additionally, the clinical factor scores allow non-clinicians to easily gain an understanding of a patient’s overall urgency relative to other patients. This information could then be used to make informed decisions on any operational changes and provide patients with an estimated waiting time based on their assigned category and clinical factor score. The DPS system also simplifies the process of handling manual interventions, such as patient cancellations or surgeon/theatre unavailability. If a patient is cancelled or delayed, they can re-join the waiting list according to their prior score, ensuring their rank on the waiting list remains reflective of their overall priority. Hence, a conclusion could be drawn that this system would perform equal or better than the current three-category system in this regard.

The findings of this research support the conclusions of other literature in the field of patient prioritisation. Specifically, in regard to the potential of patient prioritisation tools to decrease waiting time and improve admission equity through the use of consistent, reliable and transparent criteria and prioritisation methodologies that are reflective of clinical judgment [4, 24]. Additionally, this research attempted to address a gap in the literature by combining aspects of scoring tools [12, 19, 20, 25, 26], formula-based prioritisation tools [13, 14, 16, 21–23], and rigorous criteria weighting techniques [40, 41] to develop a robust prioritisation system that is scalable across a large number of procedures. In comparison to the formulations presented in Appendix A, the functional form of the proposed formulation can be seen to feature a number of similarities. For example, incorporating both the additive behaviours of traditional scoring tools and the dynamic nature of time-based formulae systems. Further, the formulation itself can be seen as logical, reasonable, and explainable, which are important characteristics to enhance clinical uptake, trust, and acceptance. This contrasts some formulations presented in Appendix A, in regard to the use of arbitrary functional forms, criterion weightings, and constant terms.

A limitation of the DPS system in regard to the clinical assessment of patients is that clinicians still need to assign an urgency category with an associated MRWT. As detailed in the Results section, MRWT (and hence assigned urgency category) is a dominating term in patients’ waiting time, which remains a discretionary decision of the treating health professional. Hence, there remains the possibility of inconsistent urgency category assignment by clinicians – for example, in the form of category creeping. However, this assignment was still required to ascertain the overall magnitude of a patient’s clinical urgency and provide an additional metric to differentiate patients. Though, the use of clinical factor selection forms may influence the practice of category creeping and reduce subjectivity by providing explicit criteria on which clinicians can base their assessments, prompting more critical consideration of urgency category assignment. In conjunction with clinical guidelines, specific category assignments may also be suggested or restricted depending on the previously entered clinical data. Therefore, in the DPS system’s current form, it should not be thought of as a complete replacement of the current system, but instead as a supplementary decision-making tool, which enhances current prioritisation practices by effectively incorporating and utilising available clinical information. Future research would focus on removing the need to select an urgency categorisation, so clinicians are only required to assess patients on explicit clinical criteria. This may involve utilising historical data to identify relationships and trends between procedures and clinical factor scores to inform on the general magnitude of a patient’s clinical urgency.

It is also noteworthy to recognise that there is no quantitative evidence supporting the treatment timeframes in the current definitions of each of the three urgency categories and were determined qualitatively by experts. Additionally, while the functional form of the prioritisation formula presented in this research is logical and reflects clinical judgment, there are an infinite number of forms it may take.

## Conclusion

This research found that prioritising patients according to the proportion of their maximum recommended waiting time (MRWT) is an effective method to improve waiting time equity, especially for patients classified as low priority, who traditionally may be waiting years for surgical intervention. Proportion of MRWT is a normalised metric across urgency categories and therefore, provides means to validly compare waiting times of patients from different urgency categories.

Consistent prioritisation methodologies were also found to contribute to waiting time equity. Prioritising patients on a predefined set of criteria through clinical factor selection forms reduces the subjectivity associated with urgency category classification. As such, better consistency can be expected for patients of similar clinical need across multiple clinicians. Additionally, by prioritisation patients according to the same set of rules (i.e., priority score), the equity and transparency of waiting list management was further enhanced.

Utilising the DPS system has significant potential to transform surgical waiting list management by incorporating explicit, defensible, and transparent criteria to prioritise surgical intervention. This system ensures that patients increase in waiting list rank at a rate aligned to their clinical need, thereby systematically improving access to elective surgery in terms of fairness and equity. The proposed priority scoring system aids in managing waiting lists by standardising waiting times across all urgency categories. While not all patients were directly affected, the DPS system improved waiting time consistency and ensured that no individual patient group were disproportionally advantaged or disadvantaged.

Under the DPS system, all stakeholders can place more confidence in the appropriateness of patients’ assigned priority. The system increases equity across all patient categories and provides consistent processes for clinicians to assess clinical need, while also including an effective and efficient means of implementation. As such, it is intended that implementation of the DPS system will increase public trust and confidence in the systems used to prioritise elective surgeries. Features of the DPS system could also be extracted to suit the needs of individual healthcare systems. For example, clinical factor selection forms may be used independently of the DPS system and could be an effective tool to aid current prioritisation practices, providing an objective metric for waiting list staff to gauge the severity of patients and better inform prioritisation decisions.

Future research would investigate techniques to remove the subjective decision of urgency category assignment from the clinical factor selection forms. This selection was required in this research to inform overall magnitude of urgency; however, future research will aim to derive MRWT explicitly from clinical factors associated with a given procedure. Additionally, as discussed above, there are an infinite number of prioritisation formula functional forms for the DPS system that exist. Future research will explore the variety of configurations of prioritisation formulae, such as the introduction of power terms, which may affect how patients progress in the system. Finally, some procedures or conditions may be inherently more severe than others and therefore, clinical factor scores from some procedures may not be directly comparable. Future research would investigate techniques to appropriately scale these scores between procedures to ensure comparability.

## Figures and Tables

**Fig. 9:**
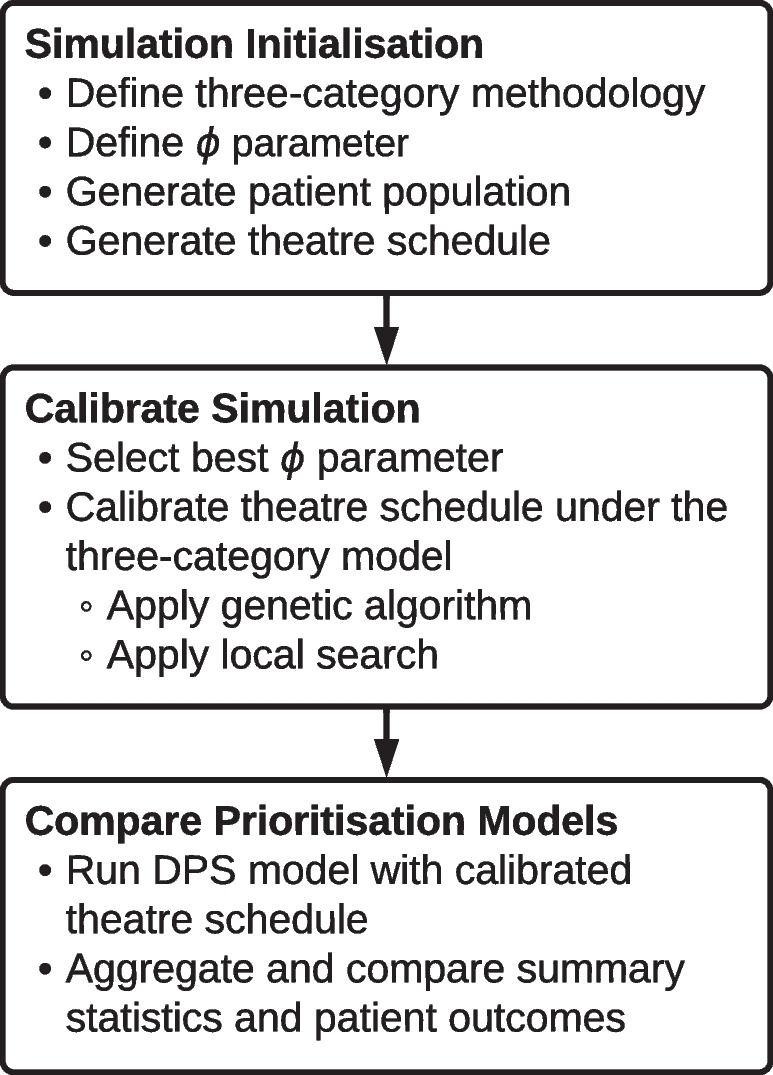
Brief overview of simulation model methodology

**Fig. 10:**
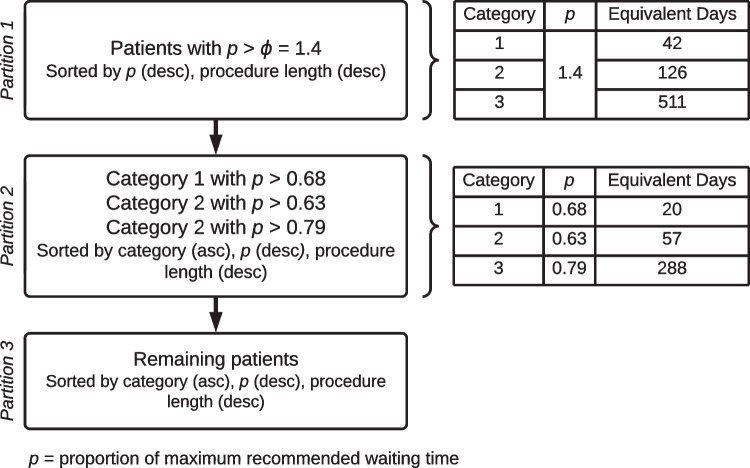
Waiting list methodology under the three-category system simulation model

**Algorithm 1:**
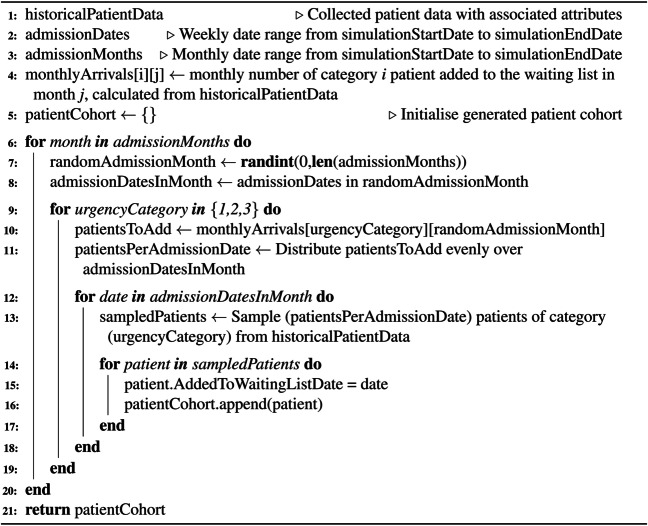
Patient cohort generation methodology

**Algorithm 2:**
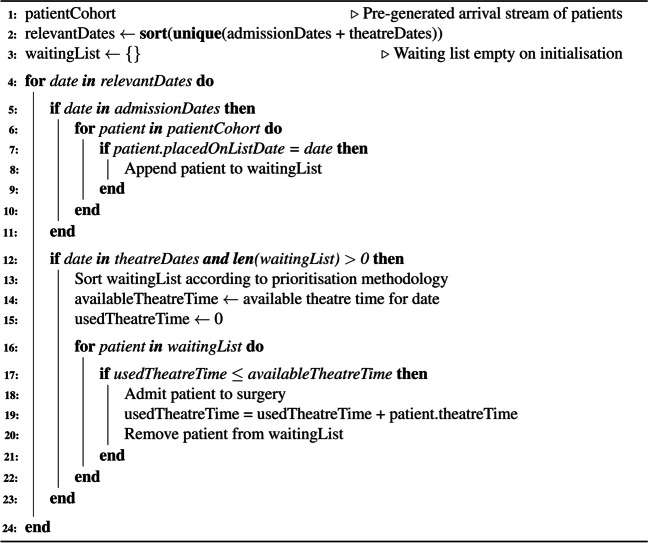
Patient admission behaviour methodology

**Fig. 11:**
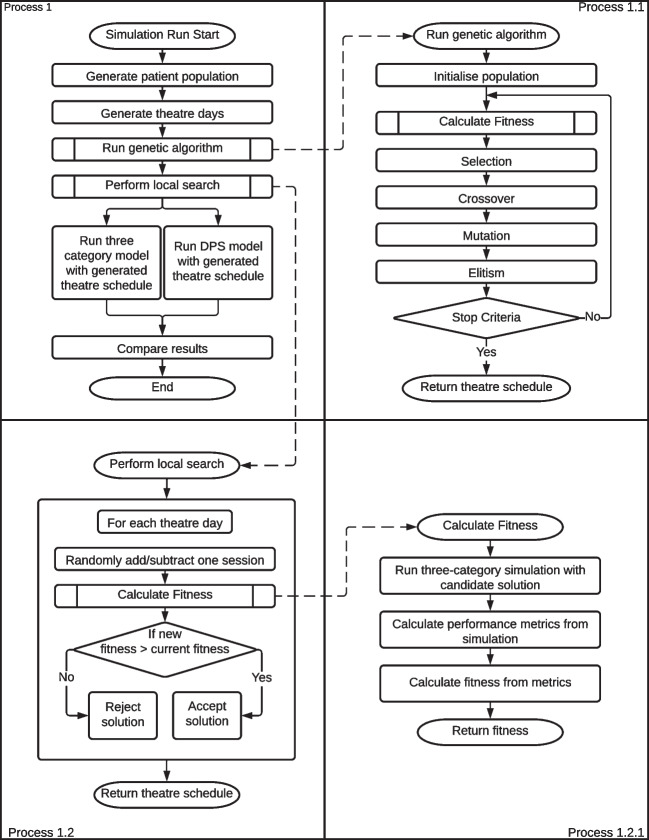
Simulation methodology for a single simulation replication

## Data Availability

Deidentified patient and clinical factor data collected and utilised during this research has been made available in a research data repository, cited in the manuscript.

## Appendix A: Selection of historical prioritisation formulations

Table 3

**Table 3:** Selection of prioritisation formulations from historical literature [47]

Reference	Proposed Prioritisation Formula	Description
[13]	$P=3{r}^{2}t+a\left(1+0.5{p}^{2}+0.5{d}^{2}\right)t$	${P}$ = priority score, $r$ = disease progression (0–4), $p$ = pain or dysfunction (0–4), $d$ = disability (0–4), $a=0 \ \text{if} \ r>0 \ \boldsymbol{ } \text{else} \ a=1$, $t$ = days waiting
[16]	$P=\frac{1}{U}(t-{t}_{0})$	$P$ = priority score, $U$ = maximum allowed waiting time for assigned urgency category, $t-t_{0}$ = time waiting
[18]	$P={\sum }_{i=1}^{n}{S}_{i}$	$P$ = priority score; $S_i$ = score for $i$ ^th^ clinical factor
[21]	$P=a\frac{w}{t}+\left(1-a\right)r$	$P$ = priority score, $a$ = priority accorded to waiting time relative to a target wait ($0\le a\le 1$), $w$ = patient waiting time, $t$ = target wait time, $r$ = random variable ($0\le r\le 1$)
[22]	$P={\sum }_{i=1}^{4}{S}_{i}^{2}{w}_{i}+{\left(\frac{5t}{m}-1\right)}^{2}$	$P$ = priority score; $S_i=$ score for $i$ ^th^ factor; $w_i=$ weight for $i$ ^th^ factor; $t$ = time already waited; $m$ = waiting time of the longest waiting patient
[23]	$P=\frac{{c}^{w}T}{\sqrt{L}}$	$P$ = priority score; $L$ = expected length of stay; $c$ = constant; $w$ = urgency or deterioration factor; $T$ = time on waiting list
[48]	$P = {S}^{a}D{T}^{{w}^{b}}$	$P$ = priority score; $S$ = social factor (1–3); $D$ = disability factor (1–3); $w$ = deterioration factor (1–3); $T$ = time on waiting list (weeks); $a$ and $b$ are constants
[49]	$AI= \left(aS\right)\left(bD\right)\left(T\right){c}^{w}$	$AI$ = admission index; $S$ = social factor; $D$ = disability factor (1–5); $w$ = urgency of condition (0–5); $T$ = time on waiting list (weeks); $a$, $b$ and $c$ are constants
[50]	$P = D{T}^{\sqrt{w}}$	Simplified version of formula proposed by [48]
[51]	$P = 800(1 -\mathrm{exp}kT)$	$P$ = priority score; $k$ = $f$(urgency); $T$ = time on waiting list
[52]	$P=\frac{{c}^{w}(T+28)}{\sqrt{L}}$	$P$ = priority score; $L$ = expected length of stay; $c$ = constant; $w$ = urgency or deterioration factor; $T$ = time on waiting list
[53]	$P=E{\sum }_{i=1}^{n}{S}_{i}$	$P$ = priority score; $E=(100-\text{age})/30$ if $\text{age}>\boldsymbol{ }70$ else $1$; $S_i=$ score for $i$ ^th^ factor

## Appendix B – Simulation design and technical implementation

A brief overview of the simulation design is initially provided in Fig. 9, with further detail of each sub-process provided in the following sections. As referenced in the aforementioned figure, $\phi$ is a control parameter of the model and is described below.

As presented in Fig. 9, the first stage of simulation design was initialisation, defining simulation parameters and model methodology. The second stage ensured simulated theatre schedules from the first stage were representative of theatre schedules used in practice under the three-category system. This was achieved through calibrating the three-category model to align with historical data and trends by matching summary statistics. Finally, the third stage ran the simulation model under the DPS system with the calibrated theatre schedule. Simulation statistics and patient outcomes between the two systems were then aggregated and compared.

### Simulation initialisation

To model the three-category system, a classical priority queue could not be used, as its behaviour is not representative of the techniques used in practice. Accordingly, to implement this model and provide an analogous comparison to current practice, priority “trigger points” and “minimum waiting times” were required to be implemented. Such constraints are not explicitly defined in reality but were implemented within the simulation model as simple rules to approximate the complex manual decision-making process waiting list staff face. This was required so that the simulation could replicate the general macro-scale system resulting from the manual process of how bookings staff manage the waiting list. For example, to manage waiting lists, patients are booked a certain number of days in advance. Though, they may be admitted sooner, delayed, or reordered on the waiting list depending on a number of operational constraints, such as the unavailability of surgical staff and operating theatres, increased surgery demand, or other clinical reasons.

This prioritisation methodology can be seen in Fig. 10, comprising three distinct partitions. The first partition captures patients waiting well past their MRWT, as determined by the control parameter $\phi$. The value of this parameter determines the proportion past their MRWT a patient should wait until they are automatically prioritised. This parameter also describes how strictly the prioritisation methodology resembles that of a classical queue. For example, setting $\phi =0$ would effectively nullify the remaining partitions and prioritise patients solely on proportion of MRWT. In practice, there is some variation in how strictly patients are prioritised after exceeding their MRWT, as admission through partition 1 should only be in extreme cases when patients are waiting well past their MRWT. Hence, a suitable $\phi$ value was required to be determined to ensure the developed prioritisation methodology was representative of prioritisation processes used in practice (discussed in Simulation Calibration below).

The second partition is where the majority of patients should be admitted through. The proportion values indicated in the figure represent the target waiting times for each of the three urgency categories, represented as a proportion of MRWT for each urgency category. These values were determined in collaboration with clinicians and waiting list staff, to reflect current practice and the approximate number of days in advance patients of each urgency category are booked for surgery. Equivalent days for each urgency category are calculated by multiplying the respective $p$ value with the MRWT for that category (e.g., MRWT for category 1 is 30 days, as described in Section 1). Finally, the third partition captures all remaining patients who did not satisfy the criteria of the previous two partitions.

To generate a patient cohort for each simulation replication, bootstrap sampling was utilised. The historical patient data were used to inform an empirical distribution of monthly waiting list arrivals for each urgency category. For each simulation month, a random historical month was chosen from this empirical distribution which informed the number of patients from each urgency category to sample. These arrivals were then distributed throughout the month so there was approximately the same number of arrivals each week. Patients in the historical data had a variety of associated attributes, such as procedure, surgical durations, clinical factors, etc. Sampling from the historical data consisted of randomly selecting a patient (with associated attributes) and appending the patient to the simulation waiting list. This process was repeated for each month in the three-year simulation horizon. The full patient cohort generation methodology can be seen in Algorithm 1.

Daily patient throughput was determined by the theatre schedule, which dictates the number of operating sessions per day. Each session had a duration of four hours. The number of patients that can be scheduled in a session is dependent on the required theatre time for each patient. Theatre time encompassed actual surgery time and changeover time, and was predetermined on commencement of the simulation. In both the three-category and DPS systems, if patients are equal in priority in any regard, prioritisation takes place in descending order of theatre time. This is to ensure that patients with long surgical durations are not disadvantaged, due to the decreased flexibility in scheduling long-duration procedures compared to those of shorter duration. The full algorithmic methodology for scheduling patients in the simulation model can be seen in Algorithm 2.

The theatre schedule is defined to be a vector of length equal to the number of surgery days in the simulation horizon. Each element represents the number of surgical sessions per day. While any theatre schedule can be generated arbitrarily, the use of such a schedule may not produce realistic simulation results. Hence, calibration was necessary to ensure that the simulation is representative of historical behaviour.

### Simulation calibration

#### Waiting list methodology calibration

To simulate theatre schedules representative of historical data, an optimal choice of $\phi$ was required to be determined. To ascertain a suitable value, a range of appropriate $\phi$ values were proposed, with the quality of each evaluated through the combination of two metrics.

The first metric was the proportion of patients admitted through each partition, as defined in Fig. 10. Admission through the first partition indicates that patients are waiting a significant proportion past their MRWT. Ideally, the number of patients waiting past their recommended treatment time should be minimised. Therefore, most patients should be admitted through the second partition, indicating that they enter surgery within or around their expected waiting time.

The second metric used was the sum of squared error of simulation waiting times across four measures (the mean, median, first quartile and third quartile), and treat in time proportions, compared to the respective historical measure for each urgency category. This ensured that the simulation model was representative of historical data and trends.

Several thousand simulation replications were conducted to determine an optimal value of $\phi$. Using the above combination of metrics, it was found setting $\phi =1.4$ (i.e., waiting 40% longer than MRWT) resulted in the best fit to the historical data.

#### Theatre schedule calibration

Similarly, in each simulation replication, the theatre schedule for each patient population and arrival stream under the three-category system must also be calibrated according to historical data and admission trends, as utilising the same predefined schedule may not result in realistic simulation behaviour. This was achieved by utilising a genetic algorithm, implemented using the Python package PyGAD [44], to evolve the optimal number of daily theatre sessions.

Genetic algorithms (GAs) are a type of evolutionary algorithm inspired by the concepts of evolution, natural selection, and survival of the fittest [45]. The algorithm starts by generating a certain number of candidate solutions, known as a population. Each candidate solution is represented by a vector of fixed length, with each vector denoted as a gene. A fitness score is used to determine the quality of each candidate solution. In each successive generation, new solutions are generated by selecting a number of parent individuals with the highest fitness values and applying perturbation operators such as crossover and mutation to generate offspring individuals. Crossover is when some random proportion of two-parent individuals are swapped to create two new offspring. Depending on the algorithm configuration, these two individuals can be added to the population or replace two inferior individuals. Mutation is when a gene value of an individual is randomly changed according to some probability. The purpose of the mutation operator is to maintain diversity in the population and prevent non-optimal convergence. After a predetermined number of iterations, a solution space of high-quality solutions will ideally be achieved [46]. The full methodology of GAs in the context of this research can be seen in Process 1.1 of Fig. 11. Readers may refer to [45] for the foundational definition of GAs [46], for a comprehensive mathematical description, and [54, 55] for further description and review of modern implementation and applications of GA.

Parameters utilised in the genetic algorithm can be seen in Table

**Table 4:** Genetic algorithm parameters

Gene type	Integer
Gene range	$\left\{\mathrm{0,1},\mathrm{2,3},\mathrm{4,5},6\right\}$
Chromosomes per population	$10$
Genes per Chromosomes	Equal to the number of operating days in the simulation horizon
Parent selection type	Steady-state selection
Parents mating	$4$
Number of parents to keep	$4$
Crossover type	Uniform
Mutation type	Random
Chance of mutation	$16\%$
Mutation gene range	$\left\{\mathrm{0,1},\mathrm{2,3},\mathrm{4,5},6\right\}$

^4^ and were determined from reviewing relevant literature and parameter optimisation techniques. Each potential theatre schedule in the genetic algorithm represented the number of daily sessions across the simulation horizon. The fitness function sought to minimise the sum of the squared error of four metrics between historical and simulated waiting times for each category by manipulating the daily number of sessions. The four metrics that were incorporated in the fitness function were the mean, median, first quartile, and third quartile of proportion of MRWT (the normalised equivalent of days waiting according to the patient’s urgency category). The specific fitness function is as follows:

$${\text{min}} \sum_{i=1}^{4}\sum_{j=1}^{3}{({S}_{ij}-{H}_{ij})}^{2},$$

where ${S}_{ij}$ is simulation metric $i$ for category $j$ patients and ${H}_{ij}$ is historical metric $i$ for category $j$ patients, such that $i=1, 2, 3, 4$ and $j=1, 2, 3$. The values of the historical metrics utilised can be seen in Table 1.

On calculation of the simulation metrics, a sufficient warm-up period was implemented before data collection started, to ensure gathered statistics were representative of normal running conditions of the simulation. Similarly, a cool-down period was implemented, characterised by data collection ceasing when the last patient was admitted to the waiting list, ensuring summary statistics were not influenced by the end of the patient stream.

During preliminary investigation, it was found that better theatre schedules (i.e., theatre schedules for a given patient stream such that simulation summary statistics matched closest to historical summary statistics) could be achieved by generating starting solutions where the total number of sessions was between a certain range. It was found that the ideal range was between approximately 225–435 sessions across the three-year simulation horizon. Hence, on the generation of each starting solution, the number of sessions in a solution was set to sum to a random uniformly distributed value between 225 and 435, utilising values from the gene range.

On completion of the genetic algorithm, a local search algorithm was implemented to further improve the evolved solution. This algorithm iterated over all genes randomly in the final solution and added or subtracted one session with equal probability. The fitness of each solution was evaluated and if a higher quality solution was produced, it would be accepted as the incumbent solution.

### Comparison of prioritisation models

To provide a high degree of confidence in the results of the simulation, approximately 2000 simulation replications were conducted (after selection of $\phi$). A single simulation replication consisted of sampling a new patient cohort across the simulation horizon and calibrating the number of theatre sessions under the three-category system. The DPS system was then run in parallel holding all else equal. The whole simulation and genetic algorithm methodology can be seen in Fig. 11.

Each simulation replication on average took approximately five hours, running Python 3.9 on an 18 core Intel Xeon Gold 6140 2.3 GHz processor with 32 GB of available RAM. However, the quality of the solutions (i.e., the generated theatre schedules for an associated patient cohort) varied dramatically, hence only the top 1% of solutions were included in the analysis. These solutions represented the simulation replications where summary statistics of the three-category simulation model were closest to summary statistics of the historical data according to the aforementioned fitness function; therefore, providing confidence in the comparability of the DPS model to historical system behaviour. These top solutions were then aggregated and compared.

## Appendix C –Clinical factor selection forms criteria weightings

Below can be seen the criteria, sub-levels, and weights that were developed for each procedure in the scope of the study, to be used in the developed clinical factor selection forms.

**Table:**

Procedure	Criterion	Mean	SD
**Breast**	**Pathology**		
	Benign	0.0%	0.00%
	Atypical/Unknown	19.84%	3.72%
	Malignant	43.24%	0.87%
	**Cavity Re-excision**		
	No	0.0%	0.00%
	Yes	18.42%	1.00%
	**Functional Consequences**		
	Nil	0.0%	0.00%
	Mild-Moderate	6.44%	1.77%
	Severe	17.07%	5.81%
	**SLN Biopsy**		
	No	0.0%	0.00%
	Yes	13.48%	7.13%
	**Other Relevant Factors**		
	Low Priority	0.0%	0.00%
	Medium Priority	4.23%	1.54%
	High Priority	7.79%	2.67%
**Cholecystectomy**	**Malignant Potential**		
	Low	0.0%	0.00%
	Moderate	22.38%	4.57%
	High	40.54%	5.46%
	**Risk of Complications**		
	Low	0.0%	0.00%
	Moderate	13.04%	3.09%
	High	23.67%	3.72%
	**Pain**		
	Nil	0.0%	0.00%
	Moderate	9.83%	2.92%
	Severe	18.31%	5.15%
	**Functional Consequences**		
	Nil	0.0%	0.00%
	Mild-Moderate	5.17%	4.94%
	Severe	9.40%	5.41%
	**Other Relevant Factors**		
	Low Priority	0.0%	0.00%
	Medium Priority	5.08%	3.56%
	High Priority	8.08%	2.96%
**Colectomy**	**Obstruction**		
	No	0.0%	0.00%
	Possible	13.13%	6.52%
	Imminent	41.94%	4.18%
	**Malignancy**		
	No	0.0%	0.00%
	In Situ/Equivocal	10.30%	4.31%
	Yes	26.05%	5.11%
	**Symptoms**		
	Mild	0.0%	0.00%
	Moderate	5.75%	1.99%
	Severe	14.17%	3.67%
	**Other Relevant Factors**		
	Low Priority	0.0%	0.00%
	Medium Priority	8.29%	7.57%
	High Priority	11.42%	7.07%
	**Functional Consequences**		
	Nil	0.0%	0.00%
	Mild-Moderate	3.65%	2.50%
	Severe	6.41%	3.40%
**Diagnostic Laparoscopy**	**Malignancy**		
	Unlikely	0.0%	0.00%
	Possible	16.50%	8.60%
	Suspected	35.55%	9.03%
	**Symptoms**		
	Nil	0.0%	0.00%
	Mild-Moderate	10.52%	5.75%
	Severe	26.34%	9.88%
	**For Staging**		
	No	0.0%	0.00%
	Yes	18.01%	1.90%
	**Functional Consequences**		
	Nil	0.0%	0.00%
	Mild-Moderate	7.39%	1.33%
	Severe	12.70%	0.29%
	**Other Relevant Factors**		
	Low Priority	0.0%	0.00%
	Medium Priority	3.70%	0.67%
	High Priority	7.39%	1.33%
**Hernia (Inguinal / Femoral / Epigastric / Umbilical / Incisional / Parastomal, etc.)**	**Risk of Complications**		
	Mild	0.0%	0.00%
	Moderate	11.78%	8.36%
	Severe	28.32%	13.86%
	**Pain**		
	Nil	0.0%	0.00%
	On Exertion	9.01%	2.95%
	All the Time	21.36%	8.77%
	**Functional Consequences**		
	Nil	0.0%	0.00%
	Mild-Moderate	5.51%	2.48%
	Severe	19.68%	8.36%
	**Reducibility**		
	Easy	0.0%	0.00%
	Difficult	10.11%	10.12%
	Irreducible	18.66%	17.94%
	**Other Relevant Factors**		
	Low Priority	0.0%	0.00%
	Medium Priority	8.07%	2.98%
	High Priority	11.98%	3.13%
**Pancreatic Resection**	**Delay may affect resectability**		
	No	0.0%	0.00%
	Yes	31.72%	3.91%
	**Malignancy**		
	Unlikely	0.0%	0.00%
	Equivocal	13.48%	0.45%
	Likely/Confirmed	28.68%	3.34%
	**Unstented Jaundice**		
	No	0.0%	0.00%
	Yes	23.09%	8.28%
	**Functional Consequences**		
	Nil	0.0%	0.00%
	Mild-Moderate	4.36%	1.28%
	Severe	8.71%	2.57%
	**Other Relevant Factors**		
	Low Priority	0.0%	0.00%
	Medium Priority	4.76%	3.02%
	High Priority	7.80%	3.59%
**Perianal (EUA / Haemorrhoids / Fistula / Abscess)**	**Sepsis**		
	Nil-N/A	0.0%	0.00%
	Intermittent	8.44%	3.10%
	Ongoing	32.65%	1.97%
	**Malignant Potential**		
	No	0.0%	0.00%
	Yes	29.52%	6.39%
	**Symptoms**		
	Minimal	0.0%	0.00%
	Moderate	9.51%	4.61%
	Severe	20.01%	1.79%
	**Functional Consequences**		
	Nil	0.0%	0.00%
	Mild-Moderate	4.19%	2.91%
	Severe	9.44%	4.33%
	**Other Relevant Factors**		
	Low Priority	0.0%	0.00%
	Medium Priority	4.19%	2.91%
	High Priority	8.38%	5.83%
**Reversal of Stoma**	**Electrolyte disturbance**		
	Nil	0.0%	0.00%
	Mild-Moderate	14.44%	12.57%
	Severe	34.72%	9.82%
	**Potential for complications**		
	Minimal	0.0%	0.00%
	Moderate	10.56%	0.79%
	Severe	27.50%	8.25%
	**Other Relevant Factors**		
	Low Priority	0.0%	0.00%
	Medium Priority	8.61%	4.32%
	High Priority	14.72%	5.11%
	**Difficulty managing stoma**		
	Minimal	0.0%	0.00%
	Moderate	6.39%	6.68%
	Significant	12.50%	5.89%
	**Functional Consequences**		
	Nil	0.0%	0.00%
	Mild-Moderate	5.28%	0.39%
	Severe	10.56%	0.79%
**Skin Lesion Excision**	**Pathology**		
	Indolent (BCC / IEC)	0.0%	0.00%
	Potentially Aggressive (SCC)	15.76%	0.53%
	Aggressive (Melanoma / Merkel / Mets etc.)	36.97%	2.11%
	**Growth**		
	Nil	0.0%	0.00%
	Stable	3.54%	0.44%
	Rapid	18.80%	6.05%
	**Location**		
	Non-critical	0.0%	0.00%
	Critical	17.37%	2.81%
	**Functional Consequences**		
	Nil	0.0%	0.00%
	Mild-Moderate	7.07%	0.88%
	Severe	17.37%	2.81%
	**Other Relevant Factors**		
	Low Priority	0.0%	0.00%
	Medium Priority	4.34%	0.70%
	High Priority	9.49%	2.54%
**Thyroidectomy**	**Compressive Symptoms**		
	Nil	0.0%	0.00%
	Dysphagia	13.57%	5.13%
	Airway	39.45%	7.46%
	**Malignancy**		
	Nil	0.0%	0.00%
	Equivocal	12.51%	5.67%
	Present	28.36%	5.63%
	**Functional Consequences**		
	Nil	0.0%	0.00%
	Mild-Moderate	5.97%	4.27%
	Severe	15.62%	11.36%
	**Other Relevant Factors**		
	Low Priority	0.0%	0.00%
	Medium Priority	3.20%	0.35%
	High Priority	8.88%	3.49%
	**Thyrotoxicosis**		
	No	0.0%	0.00%
	Yes	7.69%	1.96%
